# Command Recognition Using Binarized Convolutional Neural Network with Voice and Radar Sensors for Human-Vehicle Interaction

**DOI:** 10.3390/s21113906

**Published:** 2021-06-05

**Authors:** Seunghyun Oh, Chanhee Bae, Jaechan Cho, Seongjoo Lee, Yunho Jung

**Affiliations:** 1 Department of Smart Drone Convergence, Korea Aerospace University, Goyang-si 10540, Korea; william20000@kau.kr (S.O.); zorg0909@kau.kr (C.B.); 2 School of Electronics and Information Engineering, Korea Aerospace University, Goyang-si 10540, Korea; jccho@kau.kr; 3 Department of Information and Communication Engineering and Convergence Engineering for Intelligent Drone, Sejong University, Seoul 05006, Korea; seongjoo@sejong.ac.kr

**Keywords:** binarized convolutional neural network, gesture recognition, human vehicle interaction, sensor fusion, voice recognition

## Abstract

Recently, as technology has advanced, the use of in-vehicle infotainment systems has increased, providing many functions. However, if the driver’s attention is diverted to control these systems, it can cause a fatal accident, and thus human–vehicle interaction is becoming more important. Therefore, in this paper, we propose a human–vehicle interaction system to reduce driver distraction during driving. We used voice and continuous-wave radar sensors that require low complexity for application to vehicle environments as resource-constrained platforms. The proposed system applies sensor fusion techniques to improve the limit of single-sensor monitoring. In addition, we used a binarized convolutional neural network algorithm, which significantly reduces the computational workload of the convolutional neural network in command classification. As a result of performance evaluation in noisy and cluttered environments, the proposed system showed a recognition accuracy of 96.4%, an improvement of 7.6% compared to a single voice sensor-based system, and 9.0% compared to a single radar sensor-based system.

## 1. Introduction

As technology advances, the number of infotainment systems available in vehicles is increasing [1,2]. Car manufacturers are including more functions and services in vehicles to increase user satisfaction and provide a variety of infotainment systems and environmental control functions for the driver to manipulate [3]. Accordingly, factors that can distract driver concentration, such as setting up a global positioning system (GPS) navigation system and using an audio entertainment device have also increased [4]. However, in a road environment where various unexpected situations exist, driver carelessness can cause serious safety threats. Therefore, to control the interior of the vehicle without distracting the driver’s concentration, human–vehicle interaction (HVI) systems are becoming increasingly important [5].

Various sensors, such as touch screen, camera, depth, voice, and radar sensors are used in HVI systems for in-vehicle device control [6,7,8,9,10,11,12,13,14,15,16]. Touch screens provide the largest amount of information to users at once and show the highest recognition accuracy. However, because eyesight must remain on the touch screen for control, it can lead to dangerous situations as a result of the break in concentration on the road. In contrast, camera, depth, radar, and voice sensors can be used without causing driver neglect. However, camera and depth sensors have the disadvantages of high computational complexity, personal privacy problems, and susceptibility depending on the light condition. Because illuminance varies depending on the weather, location, and time in the road environment, it can harm the recognition performance.

Conversely, voice sensors are not affected by light environments, unlike vision-based sensors; therefore, they can be used during the day, at night, or anytime. It also requires much lower computational complexity than vision-based sensors and is inexpensive. Therefore, a voice sensor is suitable as an HVI system inside a vehicle implemented as a power-constrained and resource-constrained platform, and much research has been conducted. Voice sensor-based command recognition exhibits excellent performance without high computational costs. However, when a voice sensor is used alone, recognition performance degrades in a severely noisy environment.

Radar sensors are another solution for command recognition because they provide robust recognition regardless of the illumination environment and do not cause privacy problems. Conventionally, radar sensor-based gesture recognition uses a frequency-modulated continuous-wave (FMCW) radar to obtain a range-Doppler map (RDM) by applying a 2-dimensional (2D) fast Fourier transform (FFT). Subsequently, the feature is extracted from the RDM, and the command is recognized using a machine-learning algorithm [9,10,11]. Command classification using the FMCW radar showed high accuracy. However, the FMCW radar requires a relatively higher computational complexity and memory requirement than the continuous wave (CW) radar because of the 2D FFT. CW radars can extract micro-Doppler frequencies with less computation than FMCW radar require, which can be used as a feature for command recognition [17]. However, when a CW radar sensor is used alone in a vehicle environment, performance may be degraded in a cluttered cabin environment because of passenger movement or vehicle vibration.

Voice sensors have low computational complexity and are robust to lighting conditions, but their performance is poor in noisy environments. CW radar sensors have the advantage of requiring less computational complexity, are not affected by lighting conditions, and do not present privacy issues. However, recognition performance can be degraded in severely cluttered interiors. Therefore, if only a voice or CW radar sensor is used in a vehicle environment, command recognition performance degrades in a specific environment with either significant noise or clutter, so the sensors must be fused to complement each other.

Recently, research on deep learning algorithms for classifying fusion data has increased remarkably, and among them, convolutional neural network (CNN) algorithms have been widely used [18,19,20,21]. However, CNN algorithms are difficult to apply to power-constrained and resource-constrained platforms such as in-vehicle environments because of their high computational complexity and large memory requirements. Therefore, various studies have been conducted to implement the CNN algorithm on the embedded platform, and a popular technique for increasing resource efficiency is the quantized model [22,23,24]. Among them, the binarized convolutional neural network (BCNN) has been in the spotlight because it does not show much deterioration in performance, while dramatically reducing computational complexity and memory by reducing the parameter to single-bit precision [25,26,27,28,29].

Therefore, in this paper, we propose an HVI system that can be implemented with high recognition performance on power- and resource-constrained platforms. To ensure command recognition performance, even under environmental constraints such as illumination, noise, and clutter, we fuse the voice and radar sensor information. Furthermore, we adopt BCNN algorithms that improve CNNs’ very high computational complexity and memory requirements and can implement them on an embedded platform. The rest of this paper is organized as follows: Section 2 briefly reviews related works. Section 3 provides an overview of the proposed system, BCNN algorithms, and signal processing methods. Section 4 presents the experimental environment and performance evaluation results. Finally, the conclusions of this study are presented in Section 5.

## 2. Related Works

Recently, command classification studies with voice recognition have been conducted frequently in vehicle environments. Wang et al. [6] presented a front-end speech enhancement approach for robust speech recognition in automotive environments. In this study, a multiple Gaussian mixture model (GMM) architecture was proposed to deal with voice activity detection under low signal-to-noise ratio conditions. The trained GMMs were used to estimate the speech presence probability on a frame-by-frame basis. The estimated probability serves as basic information for the relative transfer function estimation, adaptive beamforming, and post-filtering. The average word error rate (WER) was 7.6% for several scenarios.

Loh et al. [7] implemented a speech recognition interactive system that did not disturb the driver while driving. Fourteen speech commands were transformed into the frequency domain via the discrete Fourier transform (DFT), and Mel-frequency cepstral coefficients (MFCC) features were extracted by calculating the Mel-frequency spectrum. Subsequently, the MFCC feature was used to recognize the speech command with vector quantization using the Linde–Buzo–Gray model. The accuracy of the proposed system was 78.6%.

Feng et al. [8] extracted MFCC features from speech signals, applied linear discriminant analysis (LDA), and aligned the data by applying a maximum-likelihood linear transform. Finally, maximum-likelihood linear transformation was applied to normalize the inter-speaker variability and for training and testing. The final data were combined with vehicle speed information, heating, ventilation, air conditioning (HVAC) fan status, wiper status, and vehicle type information. By adopting the hybrid deep neural network–hidden Markov model (DNN–HMM), WER was reduced by 6.3%. However, a single voice sensor system significantly reduced the accuracy in a noisy vehicle environment, and complex signal processing was required to improve performance.

Another sensor used in the HVI system is radar, and much research has focused on hand gesture recognition. Most of the studies used FMCW radar with high computational complexity. Smith et al. [9] used FMCW radar to obtain RDM information. A total of nine features, including distance and velocity information, were extracted from the RDM and used for hand gesture classification. Random forest was adopted as the classification algorithm, and the proposed system performed at above 90% accuracy for all gestures on average.

Wang et al. [10] proposed a method for continuous hand gesture detection and recognition based on the FMCW radar. RDM was acquired from raw data using a 2D FFT. A range map (RM) and Doppler map (DM) were extracted, and multiple signal classification (MUSIC) algorithms were used to extract an angle map (AM). Subsequently, hand gestures were segmented using threshold values; moreover, a fusion dynamic time warping (FDTW) algorithm was proposed, and a hand gesture recognition rate of 95.8% was achieved.

Sun et al. [11] studied gesture recognition using a radar system based on micro-Doppler information. They incorporated the range information in addition to the micro-Doppler signature to filter out undesired moving targets. They used a total of five features from the acquired data, including the number of chirp-sequence cycles and the total bandwidth of the Doppler signal. The k-nearest neighbor (k-NN) algorithm was used to classify seven gestures, resulting in an accuracy of 84%. However, as in previous studies, the hand gesture recognition system using FMCW radar requires high computational complexity and memory owing to the 2D FFT operation to acquire RDM. Furthermore, an additional clutter removal algorithm is required to improve the performance in a vehicle environment with much clutter, leading to an increase in the complexity of the system.

Because a single sensor system has limitations in some environments, two or more sensors must be used by fusion. Over the past decade, research using deep learning techniques for sensor fusion data, mostly CNN algorithms, has been ongoing. Molchanov et al. [18] presented a hand gesture recognition system that combined short-range radar, camera, and depth sensors. RDM was acquired by the FMCW radar, and distance, velocity, and angle information were obtained and then combined with the camera and depth sensors. The obtained datasets were used to learn and evaluate the 3D CNN classifier and showed a classification accuracy of 94.1%.

Münzner et al. [19] studied deep learning methods for human activity recognition (HAR). A method for optimally fusing multimodal sensors was also studied. The early fusion, sensor-based late fusion, channel-based late fusion, and shared filter hybrid fusion performances of the CNN algorithm were analyzed.

Alay et al. [20] proposed a multimodal biometric system to overcome the limitations of a single-mode biometric system. In this study, the CNN-based multimodal biometric system using iris, face, and finger vein recognition systems showed better accuracy than unimodal systems. The performance of the fusion technique was compared. The proposed system showed a performance of 99.4% with the feature-level fusion technique and 100% with the score-level fusion technique for the SDUMLA-HMT dataset [30], a multimodal biometric database. In our work, we used reasonable voice and CW radar sensors on an embedded platform for command recognition in a vehicle environment. In addition, BCNN, a lightweight CNN algorithm, was used as a classification algorithm for fusion data. To the best of our knowledge, there are no BCNN-based sensor fusion studies with voice and radar sensors applicable to embedded systems.

## 3. Materials and Methods

### 3.1. Proposed System

**Figure 1:**
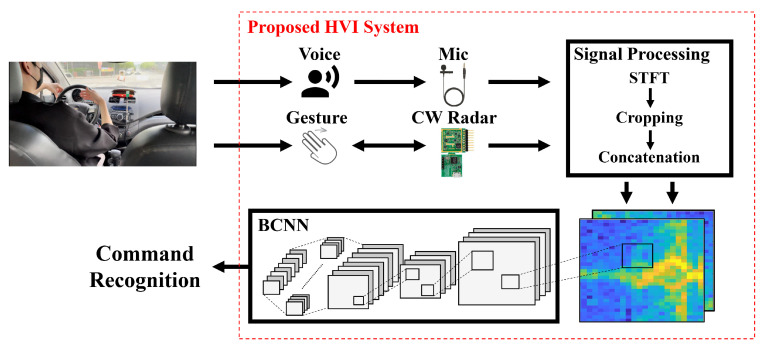
Overview of the proposed system.

As shown in Figure 1, we propose an HVI system that classifies user commands. The commands include voice commands from spoken words and gesture commands from hand gestures. The proposed system acquires voice commands using a voice sensor and gesture commands using a CW radar. We used an MVL Lavalier microphone developed by Shure [31]. The voice sensor parameters are listed in Table 1. In addition, we used a Sense2GoL CW radar made by Infineon, which has a central frequency of 24 GHz [32]. The radar parameters are listed in Table 2. Each sensor’s raw data are processed into a 2D spectrogram via a short-time Fourier transform (STFT) operation. The acquired spectrogram cropped significant signals to 32 × 24 sizes, reducing computational complexity and memory usage while matching the dimensions. The cropped 2D spectrogram was concatenated into 3D data and processed to enable BCNN learning and classification. Because voice and gesture commands should be concatenated into a 2-channel spectrogram and transferred simultaneously to BCNN, voice and radar sensor data are collected in parallel in the proposed system. Finally, the 2 × 32 × 24 sized spectrogram data were used as a 2-channel input of BCNN and processed at once to recognize the user command. Additionally, feature maps were extracted from the convolution layers (CLs). The output feature maps of the last CL were fed to the fully connected layers (FCLs), and the final class was predicted.

### 3.2. Voice Signal Processing

Voice data have many meanings and characteristics of the frequency components. Therefore, it is ideal to analyze signals through frequency conversion, rather than merely using raw data. The human voice is composed of many frequencies, and most of the voice information is concentrated below 8 kHz. According to the Nyquist sampling theory, a sampling rate of at least 16 kHz is necessary to avoid distortion of the human voice. Therefore, all voice commands were recorded at a sampling rate of 16 kHz for 1 s. When analyzing a signal whose frequency changes over time, such as a voice command, STFT can be used. Because the STFT segments a long-time signal into several short-time units and applies an FFT, it can perform analysis for each time interval. The STFT equation can be written as follows:

(1) $$X\left(\tau ,f\right)=\int_{-\infty }^{\infty }x\left(t\right)\omega \left(t-\tau \right)e^{-j2\pi ft}dt$$

where ω is the window function, and τ is the window delay time.

**Figure 2:**
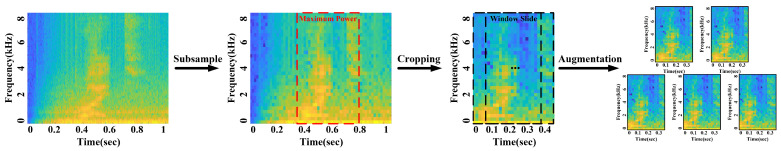
Voice signal processing flow.

**Figure 3:**
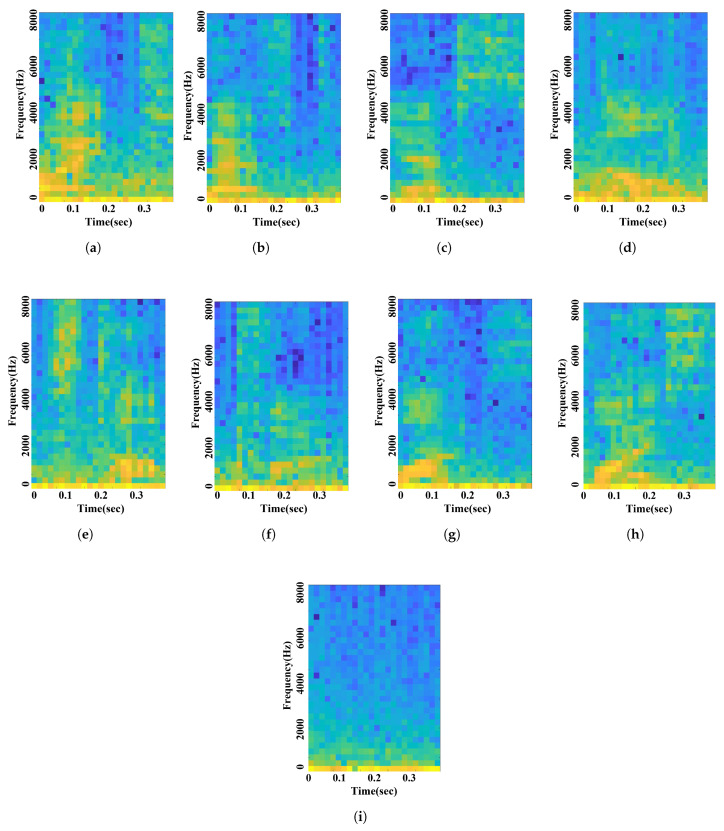
Voice spectrogram: (**a**) right; (**b**) left; (**c**) yes; (**d**) no; (**e**) stop; (**f**) pull; (**g**) once; (**h**) twice; (**i**) unknown.

A 128-point FFT and Hamming window were applied to the STFT with an overlap ratio of 50%, and a 128 × 249-sized spectrogram was obtained. The frequency axis length is 128, and the time axis length is 249. When we perform the STFT with real number data, such as voice commands, the signal is symmetrical at 0 Hz on the frequency axis, so it is reduced in size by half, and a 64 × 249-sized spectrogram is obtained. However, as the input size increases, the computation of the CNN increases exponentially, so there is a limit to using a 64 × 249-sized spectrogram as it is. Furthermore, the data cropping process is essential because the input size must be matched to the fusion with the radar data. Thus, the obtained spectrogram was 2 × 4 subsampled to obtain a 32 × 63-sized spectrogram, and the frame of maximum power signal was cropped to the size of 32 × 28. The 32 × 28-sized spectrogram is cropped into 32 × 24 and augmented into five spectrograms. The overall signal processing of voice data is shown in Figure 2. As a result, a voice data entry with a recording length of 1 second and a sampling rate of 16 kHz was processed into five spectrograms of size 32 × 24. Figure 3 represents the nine voice command spectrograms: right, left, yes, no, stop, pull, once, twice, and unknown.

### 3.3. Radar Signal Processing

CW radar can measure velocity using the Doppler effect caused by movement from the hand gesture. The Doppler effect is a phenomenon in which the frequency of the received radio wave becomes higher than the transmitted radio wave when the target approaches the radar, and the received radio wave frequency becomes lower than the transmitted radio wave when the target moves away from the radar. The change in frequency caused by the Doppler effect is called the Doppler frequency, and by the STFT of the radar signal, the Doppler frequency according to the short time change can be acquired. The gesture command used CW radar to sample 3200 data for approximately 3 s and then removed the DC offset.

**Figure 4:**
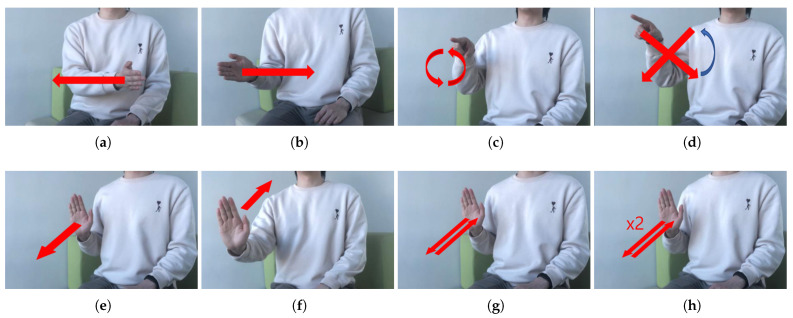
Hand gesture examples: (**a**) Hand swipe from right side to left side (symbol for right); (**b**) Hand swipe from right side to left side (symbol for left); (**c**) Hand swipe shape O (symbol for yes); (**d**) Hand swipe shape X (symbol for no); (**e**) Hand push from body to radar (symbol for stop); (**f**) Hand pull from radar to body (symbol for pull); (**g**) Hand push and pull performed once (symbol for once); (**h**) Hand push and pull performed twice (symbol for twice).

**Figure 5:**
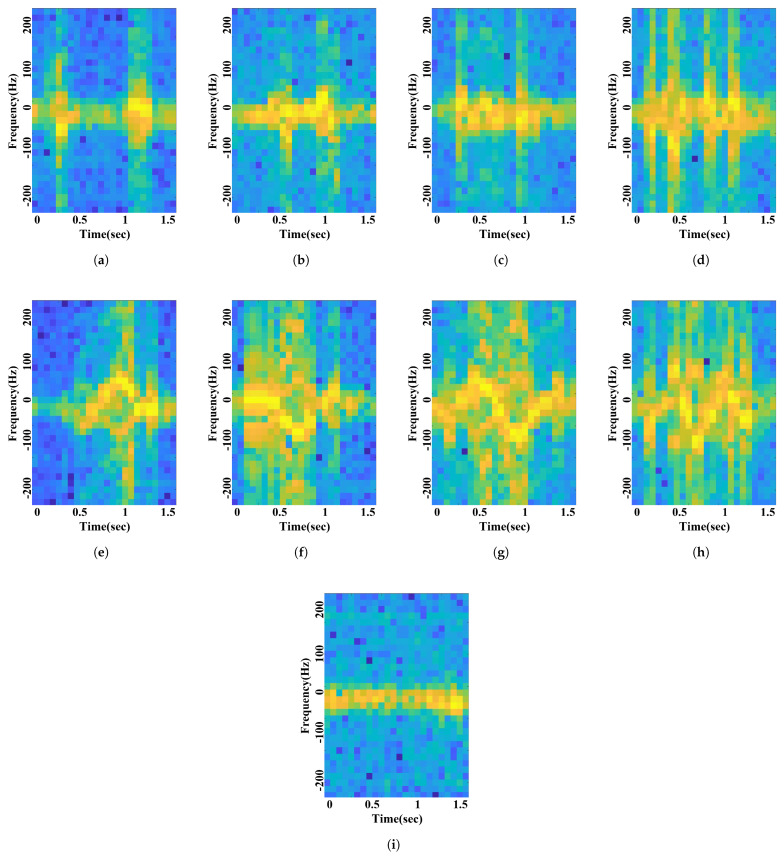
Gesture spectrogram: (**a**) right; (**b**) left; (**c**) yes; (**d**) no; (**e**) stop; (**f**) pull; (**g**) once; (**h**) twice; (**i**) unknown.

Raw radar data were processed into a 64 × 50-sized spectrogram via a 64-point STFT with a Hamming window. The maximum power signal in the time axis was cropped to 32 × 28 with 0 Hz as the center to reduce the input size and match the dimension with the voice data. The 32 × 28-sized cropped spectrogram was augmented into five spectrograms of size 32 × 24. The signal processing scheme of radar data is similar to voice. The defined nine gestures are shown in Figure 4: swipe the hand from left to right, swipe the hand from right to left, draw an “O” with the finger, draw an “X” with the finger, stretch the palm in front of the radar, pull the palm toward the body, stretch and pull the palm once, repeat stretch and pull the palm twice, and do nothing. Each of the nine class gestures means right, left, yes, stop, pull, once, twice, and unknown. The spectrograms for each gesture are presented in Figure 5.

### 3.4. Binarized Convolutional Neural Network

CNNs have the advantage of learning filters and extracting features of input data independently [33]. Therefore, CNNs do not require additional feature extraction and can use raw data without loss of information. Furthermore, owing to their robustness to distortion and changes in images, CNNs have been widely used in image classification contests and show excellent performance [34,35,36,37]. In a CNN, the CLs can extract the features via a convolution operation between the input data and the filter, and simple features such as lines are extracted from the front layer, and complex features such as texture are extracted from the back layer. The FCL uses the last output feature map of the CLs as the input and infers the final class. The pooling layer reduces the size of the data, thereby reducing computational complexity and preventing overfitting. For the pooling layer, max pooling and average pooling are commonly used. The batch normalization layer prevents internal covariate shift, improves learning speed, and solves gradient vanishing problems [38]. When it comes to the deep neural network architecture design, there exist hyper-parameter selection algorithms that have been shown to perform on par with human experts or surpassed them [39,40]. Suganuma et al. [39] attempts to automatically construct CNN architectures for an image classification task based on Cartesian genetic programming. Lorenzo et al. [40] proposed a particle swarm optimization algorithm for hyper-parameter optimization in deep neural networks. These network optimization methods show competitive results compared to state-of-the-art models. However, since most of these methods are focused on image classification, we select adequate network architecture through experiments with various network configurations. However, we think further research on network optimization is needed in the future.

CNNs have high computational complexity owing to the CLs and large memory requirements owing to the FCLs. Accordingly, much research on the weight reduction of algorithms has been conducted. Among them, the BCNN algorithm is in the spotlight [25,26,27,28,29]. The BCNN algorithm calculates the input and weight as 1 bit and significantly reduces memory and computational workload without significant performance degradation. By binarizing the CNNs, multiplication and accumulation operations for floating-point numbers can be replaced by exclusive NOR (XNOR) and pop count operations. Therefore, it is more advantageous in terms of hardware area and power, and thus it is suitable for embedded platforms. In the next section, the adequate network architecture for recognizing commands in the vehicle environment is selected, and the performance of the selected BCNN architecture is presented.

## 4. Experimental Results

### 4.1. Environment

When driving on a road, there are numerous sources of in-vehicle noise and clutter [6]. The major sources of noise that deteriorate voice recognition performance are ground-tire friction noise, engine noise, air noise when driving with the windows open, and air noise from the air conditioner or heater. The clutter types that deteriorate gesture recognition performance from the CW radar perspective are passenger movement with a Doppler frequency component and movement of an object or person due to vehicle vibration when driving on the road. In this paper, we propose an HVI system that performs classification with high accuracy, regardless of the existence of these interfering elements.

**Figure 6:**
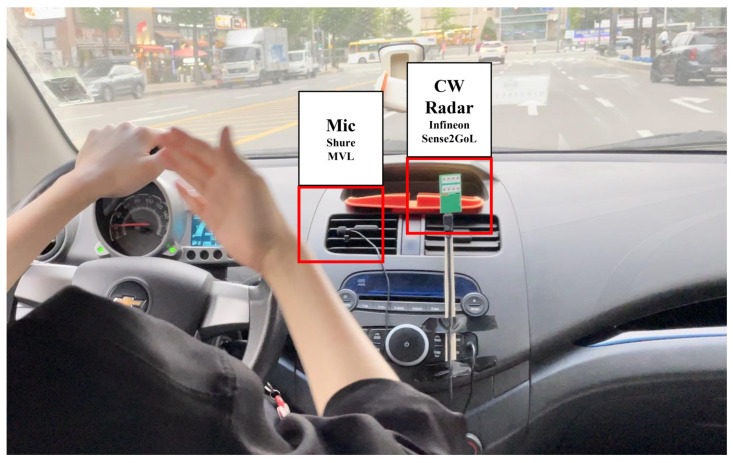
Experiment setup for voice and hand gesture recognition in vehicle.

All experiments were conducted in a real in-vehicle environment with various driving speeds ranging from 20 to 120 km/h. A total of five people participated in the experiment: two men in their 20s, one man in their 30s, and two women in their 20s. Figure 6 shows the experimental in-vehicle environment setup for acquiring data and evaluating performance. Voice commands were recorded in three environments: just driving, driving with the windows open, and driving with the air conditioner turned up to maximum. Voice commands were recorded 25 times each for one second by five participants in three environments for a total of nine commands: right, left, yes, no, stop, pull, once, twice, and unknown. Therefore, the number of raw data sets is 3375. After that, in the process of extracting the spectrogram, the data were augmented five times, so a total of 16,875 data sets were used.

The driver performed gesture commands with the right hand in three environments: no passenger, a passenger in the passenger seat and moving his upper body and arms, and a passenger sitting in the rear seat and moving his upper body and arms. As shown in Figure 6, the radar was installed in front of the center dashboard of the vehicle. The gestures were performed in the range of 30–60 cm between the driver and radar. Gesture commands were repeated 25 times each by five participants for approximately three seconds with eight hand gestures and one unknown gesture in which the driver did nothing. Therefore, the total number of raw data sets was 3375. The spectrogram was extracted, and the data were augmented five times, so the total number of data sets used was 16,875. Two 2D spectrograms extracted by voice and radar were concatenated and processed into 3D data.

### 4.2. Evaluation

**Figure 7:**
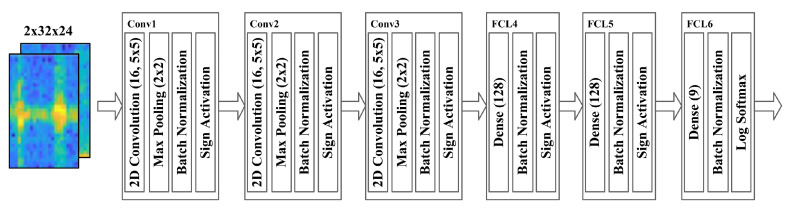
Architecture of binarized convolutional neural network.

The BCNN classifier was trained to recognize voice and gesture fusion data. All voices and gestures were normalized and used. There were 13,500 (80%) train sets used for learning and 3375 (20%) validation sets for monitoring generalization ability during training and for performance evaluation. The loss function was cross-entropy, and the optimizer was Adam for training and was trained on a GeForce RTX 2080 Ti GPU. The batch size was 200, and the epoch was 100 times. The learning rate was 0.005 before 40 epochs, 0.001 after 41 epochs, and 0.9 and 0.999 for the beta. If the network architecture is too shallow, underfitting may occur, and if it is too deep, overfitting may occur. Therefore, experiments were conducted by changing the number of CLs and FCLs to determine the adequate architecture of the BCNN. The results after evaluating the performance by changing the BCNN architecture for the fusion data are shown in Table 3. The best performance was observed with two CLs and four FCLs, three CLs and three FCLs. As shown in Table 4, both networks have similar computation times. On the other hand, the network with two CLs and four FCLs requires about 2.5 times more parameters. Therefore, we selected the network with three CLs and three FCLs. The detailed architecture of the proposed network model is illustrated in Figure 7. Each CL’s filter size was 5 × 5, the channels were each expanded to 16, and the stride was 1 × 1. Each layer’s activation function uses the Sign function to binarize the feature map to 1 for values greater than 0 and −1 for values less than 0.

**Figure 8:**
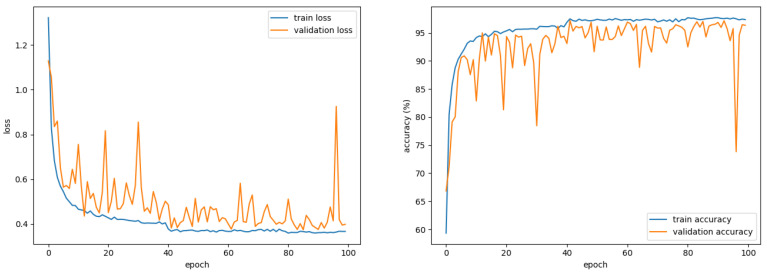
Learning curve of the 2-channel binarized convolutional neural network.

**Figure 9:**
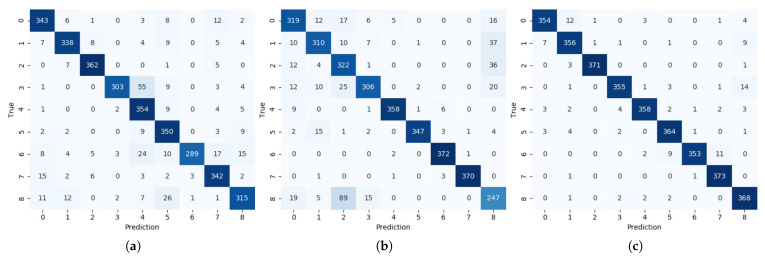
Confusion matrix of HVI system: (**a**) voice only; (**b**) radar only; (**c**) voice and radar fusion.

The proposed system classifies a command that combines a voice command with noise and a radar command with clutter in a driving environment. To confirm the effectiveness of the proposed fusion method, we presented the learning curve of the 2-channel BCNN as shown in Figure 8 and compared the performance of a single sensor system as shown in Figure 9. The single sensor system was configured identically except that the BCNN used a 1-channel spectrogram as an input. Figure 9a shows the classification results of the HVI system based on a single voice sensor as a confusion matrix. Of the 3375 datasets used for performance evaluation, 2996 were corrected, resulting in low accuracy of 88.8%. Figure 9b shows the confusion matrix for a single radar sensor-based HVI system. Of the 3375 datasets, 2951 were corrected, with a low accuracy of 87.4%. In contrast, the proposed sensor fusion HVI system, as shown in Figure 9c, showed a high accuracy of 96.4% by obtaining 3252 correct answers out of 3375. Because the CW radar only knows the approach or receding speed, gestures of right and left, yes and no, are difficult to distinguish. However, the proposed system has improved performance because it uses voice and radar sensors together. The proposed system showed 7.6% higher performance than using only a voice sensor and 9.0% higher than using only a radar sensor. The classification performance per the command of the proposed method is shown in Table 5. The proposed system showed over 94% F1 score for all commands.

**Figure 10:**
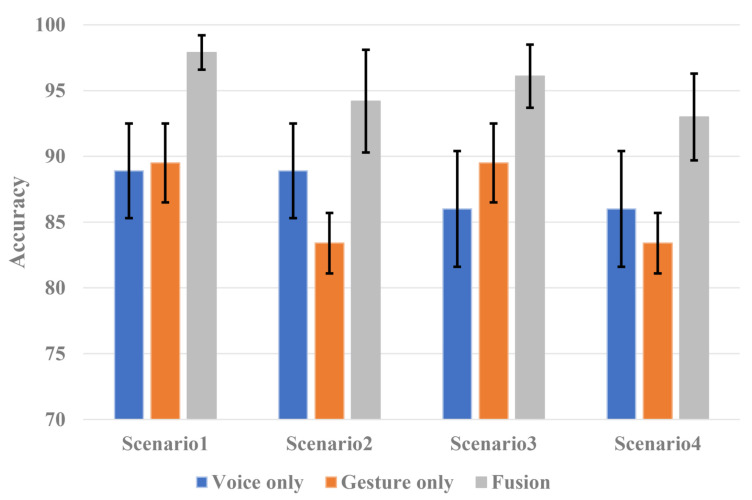
Accuracy of the HVI systems for each scenario.

In a cluttered environment where the noise is low due to simple driving but with much residual vehicle vibration or passenger movement, the voice command will show high classification accuracy, but the gesture command will show low classification accuracy. On the other hand, in a noisy environment where the clutter is low because of no residual vibration or passenger movement, but loud noise is present from opening a window or turning up the air conditioner, a low voice command classification accuracy and high gesture command classification accuracy will be observed. However, in the proposed fusion technique, two different sensors provide complementary information. Even in a limited environment for a single sensor, sufficiently good performance is observed. Therefore, to analyze the effectiveness of the proposed system, we defined good and bad datasets and developed additional scenarios to conduct the experiments. Voice commands recorded in a simple driving environment and gesture commands without passenger interference were defined as good datasets. Voice command in a noisy environment, such as opening a window or turning on an air conditioner, and gesture command with the movement of a passenger were defined as bad datasets. There were four scenarios for the experiments. The performance of the single-sensor system and the performance of the sensor fusion system were compared and analyzed for each scenario. The performance table for each scenario is shown in Figure 10.

- Scenario 1: simple driving with less noise and no movement of passengers with less clutter;
- Scenario 2: simple driving with less noise and significant movement of passengers with clutter;
- Scenario 3: opening a window or turning on the air conditioner with significant noise and no passenger movement with less clutter;
- Scenario 4: opening a window or turning on the air conditioner with significant noise and passenger movement with clutter.

The proposed HVI system has better accuracy in all scenarios, as shown in Figure 10. A single voice sensor system performs poorly in noisy environments, such as when a window is opened or the air conditioner is turned on. However, the proposed HVI system uses a radar sensor to improve its performance. The classification accuracy of gesture commands in a single radar sensor system is poor in an environment where there is significant passenger movement; however, the fusion with the voice sensor shows much better performance. Note that Scenario 4, which limited the environments for both sensors, showed low accuracies of 86% and 83.4% in the single voice sensor-based system and the single radar sensor-based system, respectively. However, in the same scenario, the proposed HVI system improved its performance by 7% compared to the single voice sensor-based system and 9.6% compared to the single radar sensor-based system with an accuracy of 93% owing to the fusion of the two sensors. Therefore, we confirm that HVI systems using the proposed fusion technique are more effective than single voice or radar sensor systems for in-vehicle environments.

We also performed k-fold cross-validation to confirm that the network generalization was successful. The four drivers’ datasets were used for training, and the rest of the datasets were used for the test. Table 6 shows the results of 5-fold cross-validation. The gesture and fusion performance of Driver 4 was a little lower because the gesture movement was relatively smaller than others due to inexperienced driving. However, in all cases, a fusion-based system is more effective than a single sensor-based system.

## 5. Conclusions

The HVI system for in-vehicle environments monitors human information based on various sensors without interfering with driving. However, it is difficult to apply a camera sensor to in-vehicle environments because of its high computational load, privacy issues, and vulnerability to dark environments. A depth sensor has the disadvantage of being vulnerable to bright light. Voice and radar sensors have the advantage of being robust in illuminated environments, and their computational complexity is smaller than that of vision-based sensors. However, if each sensor is used alone, it is vulnerable to noisy and cluttered environments. Therefore, the proposed HVI system provides robust recognition regardless of the illumination, noise, and clutter environment by fusion of voice and radar sensors, and it shows improved performance in a limited environment. CNNs can learn filters, extract features by themselves, and perform well. However, owing to the massive computation of CLs and the large memory requirement of FCLs, there is a limit to their application in embedded systems such as vehicles. Therefore, we applied BCNN algorithms using binarized inputs and weights to significantly reduce the large computational workload and memory requirements. As a result of the performance evaluation in the vehicle, a single voice sensor system showed 88.8% accuracy in a noisy environment. A single radar sensor system showed 87.4% accuracy in a cluttered environment. However, the proposed system showed a recognition accuracy of 96.4%, with a 7.6% improvement compared to the single voice sensor system and a 9% improvement compared to the single radar sensor system.

In future work, we will study a system that can more accurately classify a driver’s command by fusing voice sensors and FMCW radar sensors that can extract distance and angle information, as well as Doppler frequency. Using the FMCW radar instead of the CW radar, more information can be obtained, but the computational complexity for radar signal processing and BCNN increases. Therefore, we will study a lighter and improved signal processing algorithm and BCNN architecture. In addition, only the noise in the vehicle was considered as the interference signal of the voice sensor. In a practical driving environment, there may be two or more passengers. One can interfere with the gesture by movement, and the other can interfere with the voice by speaking. In this case, the recognition accuracy may be degraded. Nevertheless, the proposed system is expected to show better performance than a single sensor system because two different sensors complement each other. In future work, we will define additional scenarios with two or more passengers and verify the system. In addition, by applying a method of spotting voice or gesture commands, we will implement a real-time embedded system.

## Figures and Tables

**Table 1:** Voice sensor parameters.

Parameter	Value
Frequency response	45 Hz–20 kHz
Polar pattern	Omnidirectional
Signal-to-noise ratio	65 dB
Maximum sound pressure level	124 dB

**Table 2:** Radar sensor parameters.

Parameter	Value
Center frequency	24 GHz
Output power	6 dBm
Antenna gain	10 dBi
Maximum distance	15 m
Horizontal field of view	29${}^{\circ }$
Vertical field of view	80${}^{\circ }$

**Table 3:** Accuracy according to network architecture.

Convolution Layer	Fully Connected Layer			
	1	2	3	4
**1**	84.5 ± 4.5%	89.5 ± 4.5%	91 ± 3%	90.5 ± 3.5%
**2**	91 ± 3%	91 ± 3%	92.5 ± 2.5%	95 ± 1%
**3**	92 ± 2%	92.5 ± 2.5%	95 ± 1.5%	94 ± 1%
**4**	91 ± 3%	92.5 ± 2.5%	93 ± 3%	94 ± 1%

**Table 4:** Number of parameters and inference computation times.

	2CLs + 4FCLs	3CLs + 3FCLs
Number of parameters	140,256	56,320
Computation time	0.581 ms	0.622 ms

**Table 5:** Classification performance of each fused command.

	Right	Left	Yes	No	Stop	Pull	Once	Twice	Unknown
Precision	0.96	0.94	0.99	0.98	0.98	0.96	0.99	0.96	0.92
Recall	0.94	0.95	0.99	0.95	0.95	0.97	0.94	0.99	0.98
F1 score	0.95	0.94	0.99	0.96	0.97	0.96	0.97	0.98	0.95

**Table 6:** Accuracies of HVI systems for each fold.

Validation Sets	Voice Only	Gesture Only	Fusion
Driver 1	87.6%	84.4%	95.2%
Driver 2	88.3%	89.7%	97.2%
Driver 3	85.2%	83.5%	94.0%
Driver 4	86.8%	78.8%	90.9%
Driver 5	84.9%	88.6%	96.8%
